# Atorvastatin combined with dexamethasone in chronic subdural haematoma (ATOCH II): study protocol for a randomized controlled trial

**DOI:** 10.1186/s13063-021-05871-9

**Published:** 2021-12-11

**Authors:** Rong Cai Jiang, Dong Wang, Shi Guang Zhao, Ren Zhi Wang, De Zhi Kang, Xin Gen Zhu, Zong Mao Zhao, Jun Ji Wei, Ying Huang, Yan Qu, Xiao Chuan Sun, Hong Ming Ji, Xiao Chun Jiang, Jin Fang Liu, Xi De Zhu, Jian Jun Wang, Ye Tian, Chuang Gao, Hui Jie Wei, Shu Zhang, Wei Quan, Shu Yuan Yue, Ping Lei, Xian Li, Li Li Song, Craig S. Anderson, Jian Ning Zhang

**Affiliations:** 1 Department of Neurosurgery, Tianjin Medical University General Hospital, Key Laboratory of Post-trauma Neuro-repair and Regeneration in Central Nervous System, Ministry of Education, Tianjin Key Laboratory of Injuries, Variations and Regeneration of Nervous System, Tianjin Neurological Institute, No. 154 Anshan Road, Tianjin, 300052 People’s Republic of China; 2 Department of Neurosurgery, The First Affiliated Hospital of Harbin Medical University, No. 23 Youzheng Street, Nangang District, Harbin, Heilongjiang Province 150001 People’s Republic of China; 3 Department of Neurosurgery, Peking Union Medical College Hospital, No. 41 Damucang Street, Xicheng District, Beijing, 100032 People’s Republic of China; 4 Department of Neurosurgery, The First Affiliated Hospital of Fujian Medical University, No. 20 Chazhong Road, Fuzhou, Fujian Province 350005 People’s Republic of China; 5 Department of Neurosurgery, The Second Affiliated Hospital of Nanchang University, No. 1 Minde Road, Nanchang, Jiangxi Province 330006 People’s Republic of China; 6 Department of Neurosurgery, The Second Hospital of Hebei Medical University, No. 215 Heping West Road, Shijiazhuang, Hebei Province 050000 People’s Republic of China; 7 Department of Neurosurgery, Tianjin Huanhu Hospital, No. 6 Jizhao Road, Tianjin, 300350 People’s Republic of China; 8 Department of Neurosurgery, Tangdu Hospital, The Second Affiliated Hospital of Air Force Medical University, No. 1 Xinsi Road, Xian, Shanxi Province 710038 People’s Republic of China; 9 Department of Neurosurgery, The First Affiliated Hospital of Chongqing Medical University, No. 1 Youyi Road, Chongqing, 630014 People’s Republic of China; 10 Department of Neurosurgery, Shanxi Provincial People’s Hospital, No. 29 Shuangtasi Road, Taiyuan, Shanxi Province 030012 People’s Republic of China; 11 Department of Neurosurgery, Yejishan Hospital of Wannan Medical College, No. 2 West Zheshan Road, Wuhu, Anhui Province 241001 People’s Republic of China; 12 Department of Neurosurgery, Xiangya Hospital Central South University, No. 87 Xiangya Road, Changsha, Hunan 410008 People’s Republic of China; 13 Department of Neurosurgery, Linyi People’s Hospital, No. 27 Jiefang Road, Linyi, Shandong Province 276003 People’s Republic of China; 14 Department of Neurosurgery, Ordos Central Hospital, No. 23 Yijinhuoluo West Street, Ordos, Inner Mongolia Province 017000 People’s Republic of China; 15 The George Institute China at Peking University Health Science Center, Beijing, People’s Republic of China; 16 The George Institute for Global Health, Faculty of Medicine, UNSW, PO Box M201, Missenden Rd, Sydney, NSW 2050 Australia; 17 Department of Neurology, Royal Prince Alfred Hospital, Sydney, Australia

**Keywords:** Chronic subdural haematoma, Atorvastatin, Dexamethasone, Neurosurgery, Randomized controlled trial

## Abstract

**Background:**

Chronic subdural haematoma (CSDH) is a common condition in the elderly that often requires neurosurgical management. For small CSDH, evidence has emerged that statins may reduce haematoma volume and improve outcomes, presumably by reducing local inflammation and promoting vascular repair. We wish to extend this evidence in a study that aims to determine the efficacy and safety of atorvastatin combined with low-dose dexamethasone in patients with CSDH.

**Methods:**

The second ATorvastatin On Chronic subdural Hematoma (ATOCH-II) study is a multi-centre, randomized, placebo-controlled, double-blind trial which aims to enrol 240 adult patients with a conservative therapeutic indication for CSDH, randomly allocated to standard treatment with atorvastatin 20 mg combined with low-dose dexamethasone (or matching placebos) daily for 28 days, and with 152 days of follow-up. The primary outcome is a composite good outcome defined by any reduction from baseline in haematoma volume and survival free of surgery at 28 days. Secondary outcomes include functional outcome on the modified Rankin scale (mRS) and modified Barthel Index at 28 days, surgical transition and reduction in haematoma volumes at 14, 28 and 90 days.

**Discussion:**

This multi-centre clinical trial aims to provide high-quality evidence on the efficacy and safety of the combined treatment of atorvastatin and low-dose dexamethasone to reduce inflammation and enhance angiogenesis in CSDH.

**Trial registration:**

ChiCTR, ChiCTR1900021659. Registered on 3 March 2019, http://www.chictr.org.cn/showproj.aspx?proj=36157.

**Supplementary Information:**

The online version contains supplementary material available at 10.1186/s13063-021-05871-9.

## Administrative information

Note: the numbers in curly brackets in this protocol refer to SPIRIT checklist item numbers. The order of the items has been modified to group similar items (see http://www.equator-network.org/reporting-guidelines/spirit-2727-statement-defining-standard-protocol-items-for-clinical-trials/).

**Table:** 

**Title {1}**	Atorvastatin combined with dexamethasone in chronic subdural haematoma (ATOCH II): study protocol for a randomised controlled trial
**Trial registration {2a and 2b}**	Chinese Trial Registry identifier ChiCTR1900021659.
**Protocol version {3}**	V1.5; Version date:2018.7.1
**Funding {4}**	National Natural Science Foundation of China (grant 82171359, 81671380, 81971173, 82001323, 82071402), Beijing Tianjin Hebei basic research cooperation project (19JCZDJC64600) and the Tianjin science and technology program (15ZXLCSY00060, 19YFZCSY00650, 20JCYBJC00430, 20JCYBJC01380). All investigators and members of the ONET work without remuneration. The funding body will not intervene in the design of the study, analysis of the data, or writing of the manuscript.
**Author details {5a}**	^1^ Department of Neurosurgery, Tianjin Medical University General Hospital; Key Laboratory of Post-trauma Neuro-repair and Regeneration in Central Nervous System, Ministry of Education; Tianjin Key Laboratory of Injuries, Variations and Regeneration of Nervous System; Tianjin Neurological Institute, No.154 Anshan Road, Tianjin, People’s Republic of China (PRC), 300052 ^2^ Department of Neurosurgery, The First Affiliated hospital of Harbin Medical University, No.23 Youzheng Street, Nangang district, Harbin, Heilongjiang Province, PRC; 150001 ^3^ Department of Neurosurgery, Peking Union Medical College Hospital. No.41 Damucang Street, Xicheng district, Beijing, PRC;100032 ^4^ Department of Neurosurgery, The First affiliated hospital of Fujian Medical University, No.20 Chazhong Road, Fuzhou, Fujian province, PRC; 350005 ^5^ Department of Neurosurgery, Second affiliated hospital of Nanchang University, No.1 Minde Road, Nanchang, Jiangxi province, PRC; 330006 ^6^ Department of Neurosurgery, Second Hospital of HeBei Medical University, No. 215 Heping West Road, Shijiazhuang, Hebei province, PRC; 050000 ^7^ Department of Neurosurgery, Tianjin Huanhu Hospital, No. 6 Jizhao Road, Tianjin, PRC; 300350 ^8^ Department of Neurosurgery, Tangdu Hospital, The Second Affiliated hospital of Air Force Medical University, No.1 Xinsi Road, Xian, Shanxi province, PRC; 710038 ^9^ Department of Neurosurgery, The First Affiliated Hospital of Chongqing Medical University, No. 1 Youyi road, Chongqing, PRC; 630014 ^10^ Department of Neurosurgery, Shanxi Provincial People’s Hospital, No.29 Shuangtasi Road, Taiyuan, Shanxi province, PRC; 030012 ^11^ Department of Neurosurgery, Yejishan Hospital of Wannan Medical College, No.2 West ZheshanRoad, Wuhu, Anhui province, PRC; 241001 ^12^ Department of Neurosurgery, Xiangya Hospital Central South University. No. 87 Xiangya Road, Changsha, Hunan, PRC; 410008 ^13^ Department of Neurosurgery, Linyi People’s Hospital, No.27 Jiefang Road, Linyi, Shandong province, PRC; 276003 ^14^ Department of Neurosurgery, Ordos Central Hospital, No.23 Yijinhuoluo West Street, Ordos, Inner Mongolia province, PRC; 017000 ^15^ The George Institute China at Peking University Health Science Center, Beijing, PRC ^16^ The George Institute for Global Health, Faculty of Medicine, UNSW, Sydney, Australia ^17^ Department of Neurology, Royal Prince Alfred Hospital, Sydney, Australia
**Name and contact information for the trial sponsor {5b}**	Department of Neurosurgery, Tianjin Medical University General Hospital; Key Laboratory of Post-trauma Neuro-repair and Regeneration in Central Nervous System, Ministry of Education; Tianjin Key Laboratory of Injuries, Variations and Regeneration of Nervous System; Tianjin Neurological Institute. No.154 Anshan Road, Tianjin, China. Phone: + 86 22 6081 4450; Fax: +86 22 6081 4450; Email: jianningzhang@hotmail.com
**Role of sponsor and funder {5c}**	The study sponsors and funders had no role in the design, execution, analyses, interpretation of data, or decision to submit results for this study

## Introduction

### Background and rationale {6a}

Chronic subdural haematoma (CSDH) represents one of the most common forms of intracranial haemorrhage, causing a variety of diagnostic and therapeutic challenges as rates increase in ageing populations and increased use of antithrombotic agents [1, 2]. Evacuation of CSDH through twist-drill or bur-hole craniotomy remains the main form of treatment for symptomatic patients [3], but surgery is associated with high risks of operative complications and recurrence [4]. Non-surgical options include the use of mannitol, glucocorticoids, angiotensin-converting enzyme inhibitors, tranexamic acid and platelet-activating factor receptor inhibitor, but none has been shown to be clearly effective [5–8].

Impaired angiogenesis and inflammation of the surrounding neomembrane may be important in the pathophysiology of CSDH, promoting the slow expansion of blood from immature ‘leaky’ vessels after trauma [7, 9]. The pleiotropic effects of statins on inflammation and endothelial progenitor cell activity [10–14], which may promote haematoma reabsorption, were recently tested in the ATorvastatin On Chronic subdural Hematoma (ATOCH) study, a multi-centre double-blind placebo-controlled randomized trial in China [15]. The positive results of atorvastatin 20 mg daily significantly reduced haematoma volume, and improving clinical outcomes [15] has had a major impact on clinical practice in China, with this medical treatment being widely adopted for both primary conservative and adjunctive post-surgical management of CSDH [16, 17]. However, additional strategies are warranted, as response to atorvastatin is slow in many patients and ineffective over several weeks of treatment in 10% of patients [15]. Moreover, any benefits of atorvastatin at a high dose may be offset by adverse effects, such as intracranial haemorrhage [18], and may not have any greater efficacy than at low dose [13]. Dexamethasone had shown some effect on CSDH, but it is also complicated by adverse effects, particularly at high doses [19–22]. We therefore initiated the second Atorvastatin combined with dexamethasone in Chronic subdural Haematoma (ATOCH-II) trial to determine the efficacy and safety of dexamethasone combined with low-dose atorvastatin in patients with CSDH.

### Objectives {7}

#### Hypothesis

The combination of low-dose atorvastatin and low-dose dexamethasone is superior to low-dose atorvastatin alone on the outcome of treatment for patients with chronic subdural haematoma.

#### Research question

The primary aim of ATOCH-II is to determine whether the combination of low-dose atorvastatin and low-dose dexamethasone is superior to low-dose atorvastatin alone on the composite outcome of haematoma volume, transition to surgery and death at 28 days in patients with CSDH.

### Trial design {8}

ATOCH-II is a multi-centre, prospective, double-blind, placebo-controlled, randomized trial involving 240 CSDH patients with a conservative therapeutic indication for CSDH, randomly allocated to standard treatment with atorvastatin 20 mg combined with low-dose dexamethasone (or matching placebos) daily for 28 days, and with 152 days of follow-up. The demographic information of patients enrolled at each study site was imported into the central randomization system, which then generated a random number that determined whether a patient received atorvastatin or placebo at a randomization ratio of 1:1. There was no stratification during the randomization.

## Methods: participants, interventions and outcomes

### Study setting {9}

ATOCH-II trial recruited patients from 14 neurosurgery centres in China who are collaborative partners in the Oriental Neurosurgical Evidence-based Study Team (ONET). ONET is the first neurosurgical professional organization devoted to evidence-based medicine in China (Chair, Department of Neurosurgery, Tianjin Medical University General Hospital) with the mission to establish clinical research thinking, train clinical researchers, conduct randomized trials and promote the development of evidence-based medicine in neurosurgery.

### Eligibility criteria {10}

The study population will be drawn from consecutive patients with CSDH admitted to ONET neurosurgical centres. Patients are eligible who meet all of the following inclusion criteria: age ≥ 18 and ≤ 90 years; diagnosis of supratentorial (unilateral or bilateral) CSDH confirmed on CT imaging within 72 h after admission; a single unilateral haematoma ≥ 10 mL; pre-morbid functional independence, estimated according to scores 1 (no symptoms), 2 (symptoms) or 3 (some disability but independent in function) on the modified Rankin scale (mRS); the attending physician’s judgement that cerebral herniation and/or surgical evacuation are unlikely to occur; and the provision of informed consent or by an appropriate proxy according to local requirements.

The exclusion criteria include the following: known allergy to a statin (< 4 weeks), glucocorticoid or their ingredients; considered to have a high likelihood of cerebral trauma or cerebral herniation and/or requirement surgical evacuation or decompression; structural lesions including tumour, haematologic diseases, tuberculous, arachnoid cyst, vascular malformation, ventricular peritoneal shunt or other severe co-morbidity; abnormal liver function, hepatitis or uncontrolled liver disease, as well as other disease that has an influence of prognosis or the ability to assess the study outcomes; uncontrolled diabetes mellitus, with blood glucose levels consistently > 10 mmol/L; history of femoral head necrosis; recent (< 4 weeks) corticosteroid or anticoagulation use or abnormal coagulation function (< 4 weeks); likelihood of pregnancy or breastfeeding during the course of the study; participation in another clinical trial (< 4 weeks); high likelihood of poor adherence to the treatment or follow-up schedule; and any other reason according to the opinion of the attending clinician researcher.

### Consent process {26a}

Each participating site must obtain written approval(s) from their hospital research ethics committee (EC) (e.g. institutional review board [IRB]) and any other relevant regional or national body, before patient recruitment commences. For recruitment, each patient or guardian, assisted by a nurse coordinator assigned to the study, completed a short questionnaire about his or her condition, underwent physical exams by an attending neurosurgeon who was blinded to the treatment and received a CT scan. All data were collected on-site by nurse coordinators and submitted electronically to the Data Acquisition System, which also randomized the patients. All ethical and legal requirements are required to be met before any subject is enrolled in the trial. Written informed consent was obtained by the doctor from each participant or his/her legal surrogate.

### Additional consent provisions for collection and use of participant data and biological specimens {26b}

The study consent process includes permission for additional analysis of the collected data for systematic reviews and individual patient data pooling projects. Peripheral blood tests will be collected regularly to assess the health status of patients and to evaluate the safety of drug use. The protocol has been approved by the Research and Ethics Committee of the IMSS (CNIC registry number: IRB2018-088-01). The procedures are in compliance with the SPIRIT guidelines and letter of the Declaration of Helsinki, the conditions and principles of GCP, applicable local regulatory requirements and laws. Before enrolment, patients or their family guardians are fully informed of the trial, its potential outcomes and adverse events and are to provide informed consent.

### Interventions

#### Explanation of the choice of comparators {6b}

The positive results of atorvastatin 20 mg daily significantly reduced haematoma volume, and improving clinical outcomes has had a major impact on clinical practice in China. However, additional strategies are warranted, as response to atorvastatin is slow in many patients and ineffective over several weeks of treatment in 10% of patients. Dexamethasone had shown some effect on CSDH, but it is also complicated by adverse effects, particularly at high doses. We therefore initiated the second Atorvastatin combined with dexamethasone in Chronic subdural Haematoma (ATOCH-II) trial to determine the efficacy and safety of dexamethasone combined with low-dose atorvastatin in patients with CSDH.

#### Intervention description {11a}

All patients are to take oral 20 mg atorvastatin (one tablet) daily for 4 weeks as a standard of care. Those patients allocated to the intervention group will receive extra dexamethasone with a dose of 0.75 mg per once for three times daily during the first 14 days, and thereafter, the dose will be reduced to twice daily for 7 days, and then once a day for another 7 days. Thus, a total of 560 mg atorvastatin and 47.25 mg dexamethasone will be taken for 28 days. Patients assigned to the control group will receive the same open atorvastatin regimen but also placebo dexamethasone in the same schedule over 28 days.

At the end of the 28-day treatment period, haematoma volume will be re-evaluated on repeat CT scans and the following treatment regimens considered: (i) if the haematoma has resolved or present in a very small amount, the investigator can stop study treatment and only conduct follow-up observations until 180 days; (ii) if the haematoma appears reduced but still present, the investigator can decide either to consider ceasing medication and observing considering early surgery; (iii) if there is no obvious change in the haematoma, two researchers can jointly determine whether to continue conservative management or undertake surgery; and (iv) if the amount of haematoma is increased, the patient may undergo surgery.

#### Criteria for discontinuing or modifying allocated interventions {11b}

Eligible patients are centrally randomized in a 1:1 allocation ratio to intervention (atorvastatin combined with low-dose dexamethasone) and control (atorvastatin combined with matching placebo) groups using a blocked randomization method via a data acquisition system for electronic data capture (DAS for EDC, version 5.0) developed by Stemexcel Technology Co. Ltd., Beijing.

If a participant develops any form of neurological deterioration (e.g. altered level of consciousness) that is suggestive of expansion of the haematoma, they may be admitted to the hospital for assessment and treatment until their symptoms are relieved or resolve. If surgery is undertaken for a deteriorating patient, the randomized oral study medication is to be resumed as soon as possible after the operation until the end of the planned 28-day treatment period.

The patient has the right to refuse treatment at any time or to withdraw completely from the trial. The investigator can also ask the patient to withdraw from the trial, including (1) the compliance of the subjects was poor and the scheme was violated, (2) unacceptable adverse reactions/serious adverse events occur, (3) subjects experienced serious complications during the trial and urgent measures were required, (4) the subject withdraws from the clinical trial or withdraws the informed consent form and (5) other special circumstances.

#### Strategies to improve adherence to the intervention {11c}

Treatment compliance is monitored through pill counts and regular contact with participants, with instructions to adjust medication dosage at weekly outpatient clinic visits. All the patients were treated in an outpatient setting and received 7-day supplies of the medication in seven individual packages. They returned the empty packages at the end of each week to exchange them for the next week’s supply, until the treatment ended.

#### Relevant concomitant care permitted or prohibited during the trial {11d}

Most participants are treated as outpatients receiving conventional and standard cares such as rest and pain relief. Family members are responsible for monitoring the patients for progress in neurological symptoms such as worsening headaches. If a patient is reported to have worsening symptoms, he or she will be further evaluated by two attending physicians for neurological deficits. These patients may be admitted during the trial for the treatment of progressing neurological deficits and discharged when their symptoms are relieved significantly.

Within 72 h prior to treatment, CT, routine blood test, coagulation, blood glucose and biochemical analysis were rechecked before enrolling into the group, as well as neurological assessment. At the end of 2 weeks and 4 weeks, routinely check blood test, coagulation, blood glucose and biochemical analysis and re-evaluate neurological function. CT scans are performed at end of 2 weeks, 4 weeks and 3 months. mRS and ADL-BI were scored before treatment and at the end of 4 weeks, 3 months and 6 months.

Each patient receives an in-person clinical assessment at an outpatient clinic by two attending neurosurgeons who are kept blind to the treatment allocation, every 2 weeks during the 28-day treatment duration, and then each month thereafter until 180 days post-randomization.

Anticoagulants (warfarin or new oral anticoagulants) are prohibited. Antiplatelet drugs (aspirin, clopidogrel) or Xuesaitong (traditional Chinese medicine for activating blood circulation) can potentially interfere with haematoma absorption and are not recommended during the treatment. However, these patients will not be exclusively excluded from the trial participation. The following drugs may potentially interact with statins and/or cause adverse events and will not be recommended during the treatment: red yeast rice, erythromycin, fibrates, cyclosporin, macrolide antibacterials, azole anti-fungals, tacrolimus, gemfibrozil, troglitazone and itraconazole.

The use of other medications is recorded in the case report form (CRF), including their name, dose and duration of treatment. Any change in haematological and biochemical markers, such as liver function tests, and side effects, such as myalgia, constipation and other possible drug-related symptoms, will be recorded. All details of any treatment during the 180-day study period are recorded.

#### Provisions for post-trial care {30}

If a patient develops serious complications related to the experimental drugs, we will arrange for the patient to be hospitalized until the related symptoms are fully resolved.

### Outcomes {12}

The primary outcome is a composite of ‘good outcome’, including any reduction in subdural haematoma volume from baseline and free of surgery and death within 28 days of the treatment. The haematoma volume is measured centrally using the Tada formula of maximal length (L) × maximal width (W) × maximal thickness (H), divided by 2 [23], on the acquired CT images by 3 independent neuroradiologists blind to the treatment location. Details of the location, density and compartment of the haematoma are also recorded.

Secondary outcomes include functional outcome according to a ‘shift’ in the distribution of the full range of scores on mRS at 28 days; function on the modified Barthel Index measure of actives of daily living (ADL-BI) at 28 days; surgical intervention at 28, 90 and 180 days; and reduction in haematoma volume at 14, 28 and 90 days.

### Participant timeline {13}

**Fig. 1:**
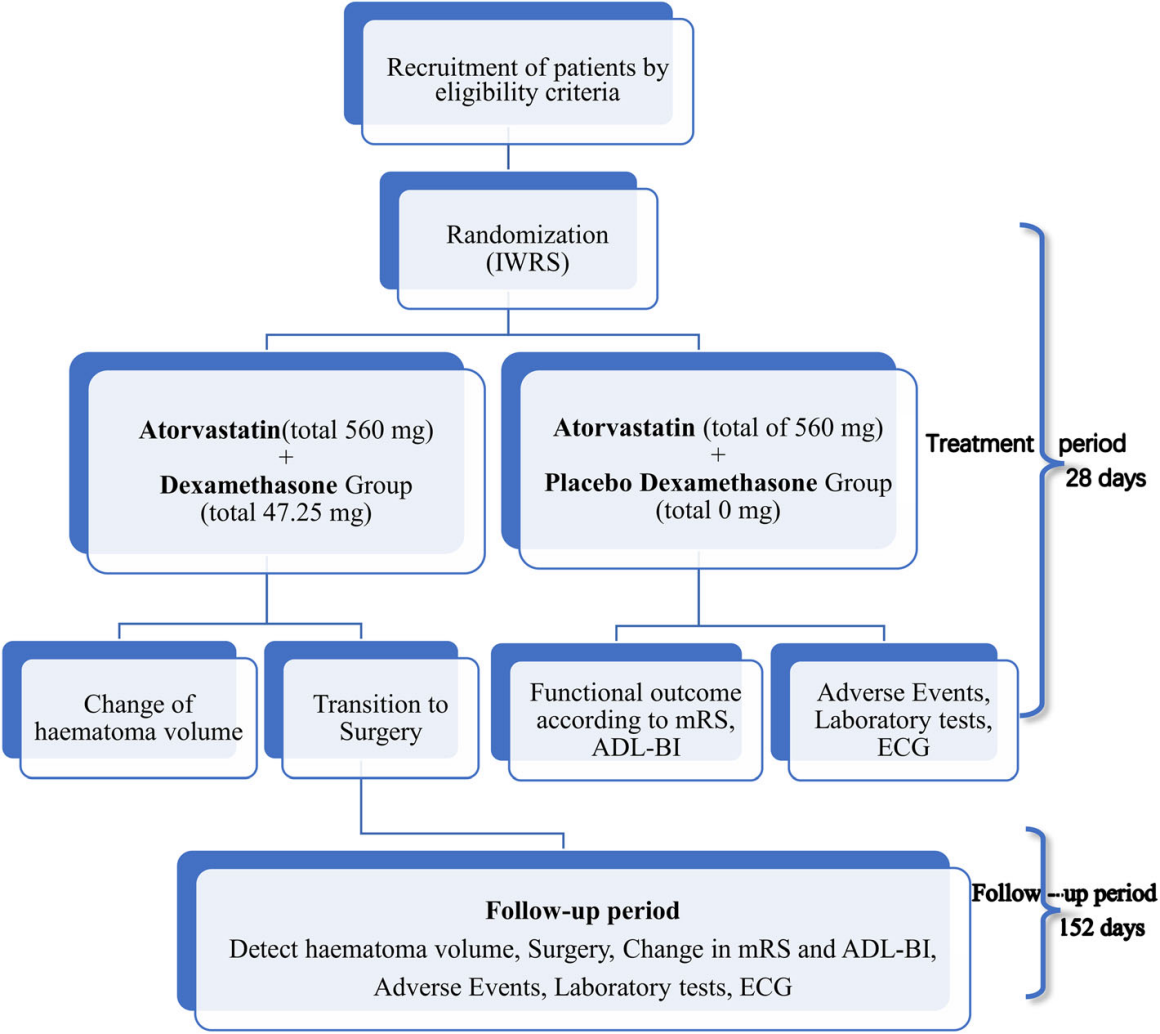
Flow chart to illustrate the design of the trial. ADL-BI, activities of daily life on the Barthel Index; ECG, electrocardiography; IWRS, Interactive Web Response System; mRS, modified Rankin scale

Baseline demographic and clinical features, including medical history, severity and type of neurological deficit and physical and biochemical results, are collected at the time of enrolment in the study. Treatment should be initiated immediately after randomization (Table 1). All background care should follow standard guideline recommendations for the management of CSDH in China which includes the withholding of anticoagulation and use of antiplatelet agents and prescribing prophylactic antiepileptic drugs and regular use analgesics. Protocol-mandated assessments will be performed according to the schedule (Table 1), and the study flow chart illustrates key steps in the trial (Fig. 1).

**Table 1:** Visit and assessment schedule

Items	Screen	Day 1	Day 7 ± 1	Day 14 ± 1	Day 28 ± 3	Day 90 ± 7	Day 180 ± 7
Informed consent	×						
Inclusion/exclusion^a^	×						
Filling general information	×						
Collect medical history	×						
Concomitant medications^b^	×	×	×	×	×		
Physical examination	×						
Surgery		×	×	×	×	×	×
Neurological symptoms^c^	×	×	×	×	×	×	×
Haematoma volume^c^	×			×	×	×	
mRS^c^	×				×	×	×
ADL-BI					×	×	×
Blood routine	×	×		×	×		
Biochemistry	×	×		×	×		
Coagulation	×	×		×	×		
Vital signs^c^	×	×	×	×	×		
Electrocardiography	×			×	×		
Adverse events		×	×	×	×	×	×
Allocate random number	×						
Drug recycling^d^	×	×	×	×	×		

*ADL-BI* activities of daily life on the Barthel Index, *mRS* modified Rankin scale

^a^Accreditation of laboratory examination within 3 days, including head CT, before enrolment

^b^Combined use of drugs before commencing study treatment and continue until the end of the 28-day treatment period

^c^During conservative treatment, if a participant’s neurological function deteriorates associated with an increase in the size of the haematoma and risk of cerebral herniation, they must change over to surgical treatment

^d^Remaining drugs are to be recycled

### Sample size {14}

We estimate a sample size of 240 patients will provide 90% power to detect a treatment effect of 20% absolute improvement in the primary composite good outcome, after considering a 5% loss-to-follow-up at 4 weeks, then 240 patients in total with 120 per group is needed to be recruited for this study and good outcome in the single atorvastatin control group of 62.6% being consistent with the single atorvastatin group of the ATOCH trial (48 of 81) [15], a pilot trial showing 20 of 30 patients had a good outcome in a single atorvastatin group [23] and medical record data showing 9 of 12 patients had a good outcome at a single centre in 2017 [17]. The combined drug treatment effect was assumed to be consistent with the percentage of good outcomes found in 29 of 30 patients in a pilot clinical trial [23] and 95 of 108 patients’ medical records from our single centre showing a good outcome.

### Recruitment {15}

All eligible CSDH patients who present to each participating hospital from the start date are prospectively and consecutively enrolled into the study.

## Assignment of intervention: allocation

### Sequence generation {16a}

Eligible patients are centrally randomized in a 1:1 allocation ratio to the intervention (atorvastatin combined with low-dose dexamethasone) and control (atorvastatin combined with matching placebo) groups using a blocked randomization method via a data acquisition system for electronic data capture (DAS for EDC, version 5.0) developed by Stemexcel Technology Co. Ltd., Beijing.

### Concealment mechanism {16b}

DAS for Interactive Web Response System (IWRS) is used to assign the randomization number to the treatment allocation and drug distribution, and each centre will compete to recruit subjects.

### Implementation {16c}

Study medication is provided free of charge to participants: atorvastatin by Pfizer Inc. and dexamethasone by Tianjin Pacific Pharmaceutical Co. Ltd. (Tianjin, China). The placebo dexamethasone, which is composed of dextrin and with the same weight and appearance as dexamethasone, is made by Tianjin Pacific Pharmaceutical Co. Ltd. (Tianjin, China), which has a certification of Good Manufacturing Practice (GMP) for pharmaceutical products issued by the State Food and Drug Administration of China.

Study medication is packed identically and labelled ‘for clinical study use only’ with a packaging number, verification code, dosage, specifications, storage, batch number, duration of usage and manufacturer. A statistician and staff independent of the study research team oversee the packaging of the study medication, with the use of a package number and drug verification code on the tag. Each participant is allocated a unique number to be applied to a drug package number for dispensing and consistency in the database.

## Assignment of interventions: blinding

### Who will be blinded {17a}

Trial participants, doctors, care providers, outcome assessors and data analysts will be blinded. After initial screening, an investigator will log onto the Central Randomization System to enter demographic information of an enrolled patient to produce a random number application. A designated individual will then apply for a drug package number and dispense the drug when the package number is consistent with the one recorded in the database. When all data were analysed completely, unblinding of experimental groups was conducted under the supervision of the Contract Research Organization and the Trial Committee.

### Procedures for unblinding if needed {17b}

All serious adverse events (SAEs) are to be reported within 24 h of the site investigator becoming aware, and a full report is to be filled to the sponsor and the State Food and Drug Administration (SFDA). The ethics committee of the pharmaceutical affairs of ONET will conduct an investigation to confirm the causes of an SAE, decide whether the participant can continue with study medication and whether the need to apply to the organization for emergency unblinding.

## Data collection and management

### Plans for assessment and collection of outcomes {18a}

Data management will be performed by the Stemexcel Technology company, Beijing, China [24], using specially designed case report forms (CRFs) on a DAS for EDC system. The table illustrates the schedule and nature of the data collection required during the study period.

### Plans to promote participant retention and complete follow-up {18b}

Treatment compliance is monitored through pill counts and regular contact with participants, with instructions to adjust medication dosage at weekly outpatient clinic visits. Each patient is given a concise diagram of the trial process with the following details: when the patient comes in to collect their medication, when and where the patient will be scheduled for tests and what the telephone number of the hospital emergency contact is.

### Data management {19}

All CRFs are first examined and verified by 2 investigators at each study site, before a data manager undertakes further central checks and confirmation or correction of any errors or inconsistencies via a clinical monitor before finalization of the data. The CRF will be inspected by an assigned person at regular intervals throughout the trial in order to verify adherence to the protocol. Data lock occurs when all subjects have completed their follow-up and there are no outstanding data queries. All computerized forms are electronically signed (by use of the unique password) by authorized study staff; all changes made following the initial entry have an electronic dated audit trail. The paper-based CRF will eventually be converted into an electronic CRF for statistical and management purposes.

### Confidentiality {27}

Every precaution is taken to respect the privacy of participants in the conduct of the study. Only de-identified data will be used for statistical analysis and the publication of results to maintain confidentiality. Only the subjects’ numbers instead of names or admission numbers can be used for identification in all the documents submitted to the sponsor. While the grouped table recording correspondence of the subjects’ names and numbers should be kept properly and confidentially by the Contract Research Organization, and not be submitted to the sponsor.

### Plans for collection, laboratory evaluation and storage of biological specimens for genetic or molecular analysis in this trial/future use {33}

Laboratory tests are carried out according to the standard blood collection procedures of Tianjin Medical University General Hospital. All specimens are not retained but are directly reported by the clinical laboratory

## Statistics methods

### Statistical methods for primary and secondary outcomes {20a}

The primary analysis will be performed according to the intention-to-treat (ITT) principle, comprising all randomized participants. A per-protocol analysis will also be undertaken for all participants who have taken at least one dose of the trial medication and complete at least one follow-up assessment. The description of indicators will be by number of cases (and percentages) for categorical variables, and mean (standard deviation) or median (interquartile range) for continuous variables. The chi-square test and logistic regression with adjustment for site, age, sex and baseline haematoma volume will be adopted for the comparison of the primary outcome between the treatment groups. For secondary outcomes, Student’s *t*-test or non-parametric Wilcoxon tests will be performed for continuous outcomes and the chi-square test for dichotomous outcomes. Ordinal logistic regression will be used for the analysis of the shift in the full range of scores on the mRS. All statistical tests are two-sided, with a *p*-value < 0.05 indicating statistical significance. All analysis will be conducted using SAS 9.4 (SAS Institute Inc., USA).

### Interim analyses {21b}

Two ‘formal interim analysis’ meetings will be held by the Data and Safety Monitoring Board (DSMB) by teleconference (or face-to-face, if possible) to review the data relating to treatment efficacy, patient safety and quality of trial conduct.

A recommendation to discontinue ATOCH-II prematurely will be based upon there being clear evidence that the treatment provides protection or causes harm for an important clinical outcome.

### Methods for additional analyses {20b}

Subgroup analysis of haematoma volume and age was planned to be conducted.

### Methods in analysis to handle protocol non-adherence and any statistical methods to handle missing data {20c}

The full analysis set (FAS) included the patients who took the drug at least once and had the primary therapeutic evaluation data at baseline. In the FAS, missing data from the 4 weeks of treatment were replaced with the last available data, in accordance with the last observation carried forward (LOCF) principle. If more than 5% of patients have a missing primary outcome, we will use multiple imputations for a sensitivity analysis.

### Plans to give access to the full protocol, participant level-data and statistical code {31c}

Datasets generated and/or analysed will be available to all study investigators, and to investigators at other institutions around the world, according to a data sharing agreement. Data sharing will be available from 12 months after the publication of the main results. We will use Research Manager (ResMan), an Internet-based public platform for clinical research electronic management (http://www.medresman.org.cn/login.aspx), for data sharing.

### Oversight and monitoring

#### Composition of the coordinating centre and trial steering committee {5d}

This trial was managed and operated by the clinical operations department of Beijing Stemexcel Technology Co., Ltd. One project manager was assigned to be responsible for the daily management of the trial, and one CRA was assigned to each participating hospital (some CRAS may be responsible for multiple participating hospitals at the same time). CRA comes to the hospital for clinical supervision once in 2 months.

Data management services are provided by the data department of Beijing Stemexcel Technology Co., Ltd. One data management manager is responsible for the daily management of trial data. At the same time, 2 database technicians are equipped to be responsible for the daily maintenance of the EDC database. Beijing SiteRegular Medical Technology Co., Ltd. provided the Clinical Research Coordinate (CRC) service for this trial. Each participating hospital was assigned a Clinical Research Coordinator (CRC), who was responsible for assisting researchers to complete various Research contents. The above members are responsible for the daily operation of the trial, and the team will hold online project meetings with the sponsor once in 2 weeks. An independent Data & Safety Monitoring Board (DSMB) regularly evaluates the progress of the clinical trial. In addition to the DSMB kick-off meeting, DSMB will convene meetings according to the following frequency: after 50% of the subjects (120 cases) are enrolled and safety observation is completed and after 75% of the subjects (180 cases) are enrolled and safety observation is completed.

### Composition of the data monitoring committee, its role and reporting structure {21a}

The project has an independent DSMB, consisting of a chair, a statistician and a clinical expert, that regularly evaluates the progress of the clinical trial, safety data and important efficacy endpoints and recommends to the sponsor whether to continue, adjust or stop the trial. All DSMB members are independent of the sponsor, DSMB members will disclose all potential conflicts of interest to the sponsor, and there are no financial interests that can influence the decision of the DSMB. The DSMB is governed by a charter outlining responsibilities, procedures and confidentiality and reviews the accumulating unblinded data at regular intervals (Additional file 1: Appendix 1).

### SAE reporting and harms {22}

A serious adverse event (SAE) is defined as an event causing or with potential to cause significant harm or requiring medical intervention, whether or not it is considered related to the study treatment. A non-serious adverse event is defined as any undesirable medical experience occurring to a patient, including abnormal laboratory values without clinical consequences.

All SAEs are to be reported within 24 h of the site investigator becoming aware, and a full report is to be filled to the sponsor and the state food and drug administration (SFDA). The ethics committee of pharmaceutical affairs of ONET will conduct an investigation to confirm the causes of an SAE, decide whether the participant can continue with the study medication and deal with the related participants’ complications and adverse events. All patients are to be followed up according to the intention-to-treat principle. The SAE should be documented in the medical records or patient file and signed and dated by the investigator, for audit and monitoring. Safety outcomes are reported to the presiding Ethics Committees in line with their requirements every 6 months, as well as for review by the DMSB at each meeting.

### Frequency and plans for auditing trial conduct {23}

All CRFs are first examined and verified by 2 investigators at each study site, before a data manager undertakes further central checks and confirmation or correction of any errors or inconsistencies via a clinical monitor before finalization of the data. The CRF will be inspected by an assigned person at regular 1-month intervals throughout the trial in order to verify adherence to the protocol. The process will be independent from investigators and the sponsor.

### Plans for communicating important protocol amendments to relevant parties (e.g. trial participants, ethics committees) {25}

The clinical trial protocol should be made and signed by researchers and sponsor together and approved by the ethics committee before testing. Any changes to the protocol arising from the implementation of the test shall be proposed by the sponsor and consulted by the multi-centre Coordinating Committee. Suggestions should be submitted in writing to the sponsor and the participating centre for signature and then restarted after approval by the Ethics Committee.

### Dissemination plans {31a}

In addition to the relevant reports developed in formats suitable for various stakeholders, the findings will be published in high impact journals, presented at national and international conferences on neurortrauma, stroke, and neurosurgery. A series of seminars will be held at the end of the study. Discussion and debate will assist in integrating the results, whatever the findings, into clinical practice and to influence the decisions of guidelines and policymakers.

## Discussion

Although there is natural re-absorption of the haematoma of CSDH [25, 26], most neurosurgeons recommend surgical evacuation in symptomatic patients despite limited randomized evidence [27]. However, the ATOCH trial has attracted considerable attention in suggesting an effective medical therapy with low-dose atorvastatin in those with mild and stable CSDH [6, 16, 17]. In China, an expert consensus statement now advocates for atorvastatin being the standard treatment for CSDH, offering reduced overall healthcare costs and avoidance of surgery. Yet, the treatment has only a modest effect over several weeks duration, and some 10% of patients fail to respond [15]. A short course of combined low-dose atorvastatin and low-dose dexamethasone may enhance the anti-inflammatory effect of either treatment alone [23].

ATOCH-II aims to extend the results of ATOCH in determining the combined effects of low-doses of atorvastatin and dexamethasone over 28 days on reduction in haematoma volume and survival free of surgery. We chose not to use change in the neurological impairment as an outcome because most patients have some change in neurological status at the time of diagnosis of CSDH and for which any temporal change can be difficult to objectively measure in a short-term open study. Conversely, the use of the mRS and ADL-BI as secondary outcomes reflects their wide use as standard measures of functional status and which reflect neurological status.

We recognize several potential concerns or limitations in this surgical trial, in particular, of selection bias introduced because of the judgement of doctors in their interpretation of the recruitment criteria in the decision to avoid early surgery. Surgery is likely to be preferentially chosen in those considered at high risk of cerebral herniation, according to contemporary practice, while patients with mild-moderate sized haematomas and mild symptoms only represent a subgroup of all CSDH. Finally, as this trial will be conducted exclusively among Chinese patients in China, there may be concerns over the generalizability of the findings to other races/ethnicities and health care systems.

## Trial status

All ethics committee approvals were granted for the study to commence across all participating hospitals in China. The study is registered on March 3, 2019 (ChiCTR1900021659). Patient enrolment commenced March 15, 2019, with version 1.5 of the protocol. As of November 7, 2021, 141 patients have been enrolled. The expected date for completion of recruitment is December 31, 2022.

## Supplementary Information

**Additional file 1.** DSMB Charter.

**Additional file 2.** Informed Consent Form.

## Data Availability

Datasets will be available to all study investigators, and investigators from other institutions around the world, according to a strict data sharing agreement. Data sharing will be available from 12 months after the publication of the main results. We will use Research Manager, an internet-based public platform for clinical research electronic management, for data sharing. Investigators are to make a formal request for data sharing through the Tianjin Medical University General Hospital, Tianjin Neurological Institute. Access will be controlled by the principal investigators, with the approval of the Trial Steering Committee.

## Abbreviations

ATOCH: ATorvastatin On Chronic subdural Hematoma
mRS: Modified Rankin scale
CSDH: Chronic subdural haematoma
ONET: Oriental Neurosurgical Evidence-based Study Team
CT: Computerized tomography
DAS: Data Acquisition System
EDC: Electronic Data Capture
IWRS: Interactive Web Response System
GMP: Pharmaceutical products
ADL-BI: Activities of daily life on the Barthel Index
SAE: Aerious adverse event
SFDA: State Food and Drug Administration
CRF: Case report form
