# Supplementary material for: Atorvastatin combined with dexamethasone in chronic subdural haematoma (ATOCH II): study protocol for a randomized controlled trial
Source: Trials. 2021 Dec 11;22:905. doi: 10.1186/s13063-021-05871-9 (PMC8666019; doi:10.1186/s13063-021-05871-9)
Supplement: Supplementary file 1 — Additional file 1. DSMB Charter. [file 13063_2021_5871_MOESM1_ESM.pdf]

Confidential

ATOCH₂

Data and Safety Monitoring Board Charter (DSMB)

A multicenter, randomized, double-blinded, placebo-controlled clinical study on the efficacy and safety of atorvastatin combined with dexamethasone in the treatment of chronic subdural hematoma

(Version No.: V1.0; Version date: July 1, 2018)

Sponsor: Oriental Neurosurgical Evidence-based Team (ONET)
Tianjin Medical University General Hospital

Signature Page for DSMB Charter

Protocol Name: A multicenter, randomized, double-blinded, placebo-controlled clinical study on the efficacy and safety of atorvastatin combined with dexamethasone in the treatment of chronic subdural hematoma

I agree and fully understand the relevant conditions listed in the Charter. I will exercise my powers in strict accordance with the requirements of the Charter, and any operation in violation of the Charter will be deemed invalid.

Signature of Data and Safety Monitoring Board Member

Lei Ping	Leiping	2018.10.7
DSMB Chairperson	Printed Name	Date

Signature Page for DSMB Charter

Protocol Name: A multicenter, randomized, double-blinded, placebo-controlled clinical study on the efficacy and safety of atorvastatin combined with dexamethasone in the treatment of chronic subdural hematoma

I agree and fully understand the relevant conditions listed in the Charter. I will exercise my powers in strict accordance with the requirements of the Charter, and any operation in violation of the Charter will be deemed invalid.

Signature of Data and Safety Monitoring Board Member

Zhao Yuanli	Yuanli Zhao	7/12/2018
DSMB Member	Printed Name	Date

Signature Page for DSMB Charter

Protocol Name: A multicenter, randomized, double-blinded, placebo-controlled clinical study on the efficacy and safety of atorvastatin combined with dexamethasone in the treatment of chronic subdural hematoma

I agree and fully understand the relevant conditions listed in the Charter. I will exercise my powers in strict accordance with the requirements of the Charter, and any operation in violation of the Charter will be deemed invalid.

Signature of Data and Safety Monitoring Board Member

Jinghua Wang	Jinghua Wang	July 12, 2018
DSMB Member	Printed Name	Date

Contents

Signature Page for DSMB Charter........ 2
Signature Page for DSMB Charter........ 3
Signature Page for DSMB Charter........ 4
Contents........ 5
1 Background........ 6
2 Purpose........ 6
3 Membership........ 7
3.1 DSMB members........ 7
3.2 Financial disclosure and conflict of interest........ 8
3.3 DSMB support group........ 8
4 Responsibilities........ 8
4.1 DSMB members........ 8
4.2 DSMB support group........ 9
4.3 Sponsor........ 10
5 DSMB principles........ 10
5.1 Independence........ 10
5.2 Confidentiality........ 11
6 Meetings........ 11
6.1 DSMB legal attendees........ 11
6.2 Meeting format........ 11
6.2.1 Open session........ 12
6.2.2 Closed session........ 12
6.3 Meeting frequency........ 12
6.4 Conditions for holding an emergency meeting........ 13
7 **DSMB meeting materials and data**........ 13
7.1 Meeting communication........ 13
7.2 Meeting materials........ 13
7.3 Data sources........ 13
7.4 Data locking point........ 14
7.5 Processing of data report in paper form........ 14
7.6 Meeting minutes........ 14
8 DSMB Recommendation Letter........ 14
9 Confidentiality........ 15
9.1 Data disclosure........ 15
9.2 DSMB communication........ 15
9.3 Communication with regulatory departments........ 15
Appendix 1: Contact Information for DSMB Members........ 17
Appendix 2: Contact Information for DSMB Support group........ 18

1 BACKGROUND

In order to safeguard the interests of patients and ensure the objectivity of data, the Data and Safety Monitoring Board (hereinafter referred to as DSMB) was established for the " multicenter, randomized, double-blinded, placebo-controlled clinical study on the efficacy and safety of atorvastatin combined with dexamethasone in the treatment of chronic subdural hematoma" carried out by the Oriental Neurosurgical Evidence-based Team (ONET) and the Tianjin Medical University General Hospital to analyze and review the study-related data.

2 PURPOSE

The Charter mainly describes the procedures followed by DSMB in reviewing the study-related data as follows. DSMB provides professional consultation to the sponsor independently to protect the interests of the subjects and evaluate the safety of the study during the trial.

The specific operations of DSMB for safety evaluation in this study are as follows:

After 50% of the subjects (120 cases) are enrolled and safety observation is completed: DSMB reviews the safety data for 50% of the subjects according to the protocol and evaluates the safety of the study.

After 75% of the subjects (180 cases) are enrolled and safety observation is completed: DSMB reviews the safety data for 75% of the subjects according to the protocol and evaluates the safety of the study.

According to the protocol, if any of the "criteria for trial discontinuation" is met during the clinical study, the trial shall be suspended first, and the DSMB meeting shall be held to investigate the safety, analyze and determine whether to continue the trial.

The criteria for trial discontinuation are as follows:

(1) Serious safety concerns occurred during the trial;

(2) The sponsor requested discontinuation.

In case of any of the above conditions, the trial shall be suspended first, and the DSMB meeting shall be held immediately to investigate the safety, analyze and determine whether to continue the trial.

DSMB shall provide relevant recommendations to the sponsor according to the discussion results at the meeting, and the sponsor shall finally decide to accept or reject DSMB's recommendations. All decisions shall comply with Good Clinical Practice (GCP), *Helsinki Declaration* and relevant national or international regulations to ensure the maximum safety and interests of subjects.

3 MEMBERSHIP

3.1 DSMB members

On the basis of the opinions solicited from the principal investigator, the sponsor selects three experts to form the DSMB. DSMB consists of 2 clinical trial experts and 1 statistical expert. All members shall share the following consensus: it is the basic right of all members to know the progress of DSMB meetings and related activities, which must be kept strictly confidential. DSMB members shall not have any conflict of interest with this study. See Section 3.2 of the Charter for a detailed description of the conflict of interest.

The sponsor elects a chairperson among the DSMB members. Once one of the members cannot continue to assume responsibilities in the DSMB, the DSMB chairperson will submit a replacement to the sponsor. If the chairperson cannot continue to assume responsibilities, the sponsor will re-appoint another member as the chairperson, and then the new chairperson will submit his own replacement to the sponsor.

All the above personnel have relevant professional knowledge background, have participated in many clinical trials, are familiar with the clinical study process, and have no major conflict of interest with this study. The existing DSMB members are as follows, and the complete contact information for each member is shown in Appendix 1.

**Name**	**Position**	**Affiliation**	**DSMB role, professional field**

3.2 Financial disclosure and conflict of interest

DSMB members will disclose to the sponsor all potential, existing or perceived conflicts of interest as well as the financial interest issues that will not affect DSMB's decision-making. All members shall be completely independent individuals other than investigators. In addition to the remuneration for DSMB services, they shall have no conflict of interest with this trial in economic, scientific research or other aspects. They shall disclose the economic benefits from the sponsor. In addition to the remuneration for services, DSMB members shall not receive the consultation fees from the sponsor.

In addition, DSMB members cannot directly participate in the trial design, be involved in other study topics within this study plan as a principal investigator, investigator or competent physician, or join in any similar trials organized by other sponsors at the same time.

However, some activities of DSMB members can be considered as not a conflict of interest, such as participating in clinical study of other projects of the sponsor, previously joining in consultation work in other projects of the sponsor, etc.

3.3 DSMB support group

DSMB support group provides analysis results for DSMB data review meeting. To ensure the accuracy of the study, the support group includes an independent data management personnel and a DSMB secretary. The meeting materials, meeting charter writing and meeting preparation shall be undertaken by the staff designated by the chairperson of DSMB.

The complete contact information for DSMB support group members is shown in Appendix 2.

4 RESPONSIBILITIES

4.1 DSMB members

As a part of this study, DSMB is responsible for independently reviewing the analysis and statistical report of the study, so as to further protect the interests and safety of subjects. The main task of DSMB is to make recommendations based on the analysis results of accumulated data at each stage.

All DSMB members are required to perform the following duties and obligations:

- Understanding the trial protocol of this clinical study project in advance;
- Participating in meetings of the independent monitoring committee;
- Reviewing and signing the Data and Safety Monitoring Board Charter of the clinical trial;
- Evaluating the safety of the study according to the safety analysis report, and participating in making corresponding decisions.

The **DSMB chairperson** also has the following responsibilities: presiding over the DSMB meeting, summarizing the recommendations of DSMB members on the project, and conveying the recommendations at the meeting (in oral or written form or via e-mail) to the sponsor.

4.2 DSMB support group

Data management: In order to ensure the accuracy and impartiality of the study results, the data management personnel of DSMB support group will process the data according to the cleaned database provided by the sponsor and submit it to the independent statistician for analysis (mainly data summary and descriptive analysis). The analysis plan will be distributed to members before DSMB decision-making meeting. The independent statistician shall keep all analysis results confidential until the end of the study, and these results can be recorded in the final study report as supporting documents for statistical analysis.

Meeting secretary: Preparing and managing relevant materials (meeting minutes, communication letters, resumes, contracts, reports, recommendation letters and other documents) for DSMB, writing the draft charter, contacting the database for information transmission, and obtaining and summarizing the information of all parties. Managing corresponding logistics support and conference affairs for the smooth convening of DSMB meetings.

Clinical expert: Providing clinical opinions on data processing and statistical analysis before DSMB decision-making meeting, and playing a role of a DSMB candidate member. In case of vacancy

of a DSMB member, the clinical expert shall assume the responsibilities of DSMB member upon approval by DSMB chairperson and/or the sponsor.

Logistics personnel:

Liaison:

1) Liaison of DSMB Committee: On behalf of DSMB, the liaison is responsible for contacting the sponsor to communicate and negotiate with the sponsor on some logistics support, conference affairs and other cooperation information, and ensuring the security and confidentiality of documents during transmission.

2) Liaison of the sponsor: On behalf of the sponsor, the liaison is responsible for contacting the DSMB liaison to convey the wishes of the sponsor and put forward opinions on logistics support and conference affairs, providing the transmission of DSMB related documents and data and ensuring the security and confidentiality.

4.3 Sponsor

1) Providing DSMB with relevant documents required to perform its functions:

- Study protocol of the project
- Case Report Form
- Reviewing the statistical analysis plan provided by the independent statistician

2) After reading the recommendations put forward by DSMB, if any objection to such recommendations was raised, the sponsor will summarize DSMB's recommendations and its own comments and provide them to the ethics committee.

5 DSMB PRINCIPLES

5.1 Independence

In the process of review, evaluation and decision-making, it is necessary to maintain sufficient independence. It is intended to ensure that DSMB will review the evaluation data without any external

influence and bias and make objective decisions. It is also helpful to maintain the confidentiality of statistical analysis results.

5.2 Confidentiality

As the only organization that has access to the analysis results before the end of the study, DSMB shall ensure the confidentiality of the review contents and results, and the statistical analysis results shall be kept confidential to the sponsor and investigators in particular. No one except DSMB members or statistical experts and independent statisticians who conduct these analyses and submit them to DSMB can obtain the results of statistical analysis.

DSMB members shall sign confidentiality agreement and financial disclosure statement before reviewing study data and results.

6 MEETINGS

6.1 DSMB legal attendees

Three members of DSMB are legal attendees, and the meeting attended by all three members is considered as a valid meeting.

6.2 Meeting format

Data review meeting consists of the following defined parts. The trial protocol shall be submitted to DSMB before the DSMB kick-off meeting. DSMB members shall be familiar with the basic situation of the trial before the DSMB kick-off meeting. The sponsor shall keep DSMB informed of any revision of the study content in a timely manner. Except for the kick-off meeting, other meetings have two formats: open session and closed session. Relevant experts and the sponsor can be invited to the open session, and only DSMB members and DSMB special invitees can participate in the closed session.

Generally, face-to-face meetings are used. However, in special cases, if face-to-face meetings are not feasible, or when DSMB has held several meetings and all DSMB members are very familiar with

the trial and the problems analyzed, telephone or network video meetings can also be acceptable, but the confidentiality of the meeting shall be paid attention to.

6.2.1 Open session

The investigators, the sponsor and DSMB members participate in the open session where the sponsor can tell the DSMB members about the research implementation process, introduce the research background information and answer their questions. The data that can be discussed in the open session include enrollment, baseline characteristics, exclusion, data management, etc. The meeting secretary in the DSMB support group is responsible for the meeting minutes. All attendees shall be listed in the meeting minutes, which shall be kept by the DSMB support group until the end of the study.

6.2.2 Closed session

Only DSMB members and DSMB special invitees can participate in the closed session. In the closed session, the data of this study are reviewed and widely discussed, and after extensive discussion, written recommendations are given. At the same time, the independent statistician or the academic secretary of the DSMB support group shall take minutes of the meeting, and all attendees shall be listed in the minutes. The meeting minutes shall be kept by DSMB until the end of the study.

6.3 Meeting frequency

In addition to the DSMB kick-off meeting, DSMB will convene meetings according to the following frequency. If the conditions for holding an emergency meeting are met, the DSMB emergency meeting shall be held as soon as possible. The kick-off meeting shall be in the format of open session, the subsequent meeting shall be in the format of open session or closed session, and the format of emergency meeting shall be jointly agreed by DSMB members according to actual needs.

According to the study protocol, the meeting frequency of this study is:

① After 50% of the subjects (120 cases) are enrolled and safety observation is completed:

② After 75% of the subjects (180 cases) are enrolled and safety observation is completed:

6.4 Conditions for holding an emergency meeting

In case of any of the following situations during the trial, an emergency meeting shall be held:

Trial suspension criteria:

(1) Serious safety concerns occurred during the trial;

(2) The sponsor requested discontinuation.

7 DSMB MEETING MATERIALS AND DATA

7.1 Meeting communication

The independent statistician shall send the safety summary report to DSMB members via encrypted mail. DSMB members shall fill in the report and reply with corresponding comments after reading the report, and discuss it at the DSMB meeting.

7.2 Meeting materials

Documents and materials related to the meeting (including the Protocol and Statistical Analysis Plan) shall be delivered to the DSMB meeting secretary by express or e-mail within 7 working days before the meeting, so that DSMB members can have enough time to review. The materials will be transmitted by means of electronic encryption protection.

7.3 Data sources

The sponsor or the designated data management organization of CRO coordinates data management according to the SOP of data management. All study-specific treatments and regulations shall be recorded by the data management department. Data on serious adverse events are provided by the sponsor.

When the data is handed over to the independent statistician, the SOP related to data transfer shall be observed and the data shall not be disclosed to others. For serious adverse events, data transfer shall

be completed at the first time. The summary of other safety indicators shall be transferred to the DSMB independent statistician before the DSMB regular meeting.

7.4 Data locking point

The data used for DSMB review shall have been cleaned and frozen. The data locking point shall be the time before the data management department hands over the data required for DSMB review to the independent statistician.

7.5 Processing of data report in paper form

After the DSMB meeting, collect all DSMB data reports in paper form distributed to DSMB members, keep two copies for archiving, and destroy the rest. One is kept by the DSMB statistician and the other by the DSMB chairperson in a secure document.

7.6 Meeting minutes

The minutes of each meeting shall be kept and released to the sponsor and DSMB members within 7 working days after the meeting. The minutes shall not contain any information of data.

8 DSMB RECOMMENDATION LETTER

On the day of the closed session, the DSMB chairperson will send the first draft of the Recommendation Letter to all DSMB members for discussion. The DSMB chairperson will finally complete the DSMB Recommendation Letter after all DSMB members agree and within 7 working days after the closed session.

DSMB shall express its recommendations to the sponsor very clearly and accurately. The DSMB Recommendation Letter mainly includes considerations in safety and study execution and will observe the guiding principles in the Charter. The Recommendation Letter can provide a small amount of clear data for the sponsor to make a reasonable decision on the recommendations. The DSMB

Recommendation Letter shall include the date of the meeting, the place of the meeting, the status of the analysis data, the recommendations of the DSMB Committee, and the signatures of the DSMB Committee and the signing date. The recommendations mainly are as follows: (1) the clinical study can be continued; (2) the clinical study can be continued after the study protocol is modified; (3) the clinical study shall be suspended.

The DSMB chairperson will finally send the the DSMB Recommendation Letter to the sponsor via encrypted mail.

9 CONFIDENTIALITY

After signing the DSMB Charter, each DSMB member undertakes to abide by the provisions on data release and confidentiality set out in this Charter.

9.1 Data disclosure

Only DSMB members have access to the study results before the sponsor publishes the study results. Once any DSMB member divulges data before the end of the study, the position of one or all of DSMB members will be immediately dismissed.

9.2 DSMB communication

In addition to the exceptions specified in the Charter, it is not allowed to conduct any discussion on DSMB agreement and DSMB Recommendation Letter outside DSMB meeting, whether oral or written communication. Unless otherwise specified in the Charter, the study results shall be kept strictly confidential and shall not be disclosed to anyone other than DSMB members until the proposal to publish the results is accepted and takes effect.

9.3 Communication with regulatory departments

DSMB’s recommendation letter sent to the sponsor is only to provide suggestions, and the sponsor can decide whether to accept them or not. For any written results given by DSMB, the sponsor shall

inform DSMB before submission to the regulatory agency or other health organization.

APPENDIX 1: CONTACT INFORMATION FOR DSMB MEMBERS

Name	Position	Contact number	Mailbox	Affiliation	DSMB role, professional field
Lei Ping	Professor	13802066206	leiping1974@163.com	Tianjin Medical University	DSMB chairperson, clinical expert
Zhao Yuanli	Professor	13801121203	zhaoyuanli@pkuih.edu.cn	Beijing Tiantan Hospital, Capital Medical University	DSMB member, clinical expert
Jinghua Wang	Professor	13920672316	jhw8799@163.com	Department of epidemiology, Institute of Neurology, Tianjin Medical University	DSMB member, statistician

APPENDIX 2: CONTACT INFORMATION FOR DSMB SUPPORT GROUP

Name	Position	Contact number	Mailbox	Affiliation
**Tian Hengli**	**Professor**	**13621663102**	**tianhlsh@126.com**	**Shanghai Sixth People's Hospital Affiliated to Shanghai Jiao Tong University**
**Ge Xintong**	**Liaison/Secretary of DSMB Committee**	**13803071942**	**gexintongbob@163.com**	**Tianjin Medical University General Hospital**
**Tian Ye**	**Liaison of the sponsor**	**13672031439**	**tianye030710@163.com**	**Tianjin Medical University General Hospital**
