# Supplementary material for: Atorvastatin combined with dexamethasone in chronic subdural haematoma (ATOCH II): study protocol for a randomized controlled trial
Source: Trials. 2021 Dec 11;22:905. doi: 10.1186/s13063-021-05871-9 (PMC8666019; doi:10.1186/s13063-021-05871-9)
Supplement: Supplementary file 2 — Additional file 2. Informed Consent Form. [file 13063_2021_5871_MOESM2_ESM.pdf]

**Efficacy and safety of atorvastatin combined with dexamethasone in the treatment of chronic subdural hematoma: a multicenter, randomized, double-blind, placebo-controlled clinical study**

# Informed Consent Form

Version number/version date:V2.0/ 11 JUL2019

| | | |
|---|---|---|
| **Screening No.** | **:** | □□□□ |
| **Subject Name** | **:** | ______________________________ |
| **Contact address** | **:** | ______________________________ |
| **Contact number** | **:** | ______________________________ |
| **Center name** | **:** | ______________________________ |
| **Center number** | **:** | ______________________________ |
| **Name of Investigator** | **:** | ______________________________ |
| **Name of sponsor** | **:** | **Oriental Neurosurgical Evidence-based Team, TIANJIN MEDICAL UNIVERSITY GENERAL HOSPITAL** |

# Content

## 1. Why do I sign the informed consent?

Before you decide to participate in the research, you should understand the purpose of the research, what is the study about, how your data will be used, possible benefits, risks and discomforts.

This informed consent contains information that will help you decide whether or not to participate in the study. Please take the time to read the following carefully, and if there's anything you don't understand, please consult with your research physician or investigator. You may not sign this informed consent until you have understood all the information contained in it and have received satisfactory answers. If you are participating in other drug clinical study, you are not eligible for this study.

As one of investigators of the study, your physician will be very concerned about your health.

## 2. What are the background and object of the study?

Chronic subdural hematoma (CSDH) is one of the common intracranial hematomas in neurosurgery. Clinical symptoms and signs are usually discovered after 21 days (3 weeks) of head injury. The current treatment for CSDH is focused on borehole hematoma drainage, but the surgery carries a recurrence rate of 3%~20% with any treatment. Some CSDH are self-healing during routine follow-up, but there is an increased risk of hematoma. Our previous studies have confirmed that oral atorvastatin calcium tablets promote the absorption of hematoma. Many patients receive atorvastatin calcium for treatment and the hematoma becomes smaller or even disappears, thus avoiding surgical risks and reducing the medical burden. However, our previous study mainly focused on patients with small hematoma, and there are still cases where the hematoma continues to grow and lead to surgery. Therefore, it is necessary to develop optimized drug treatment methods to further improve the efficacy and expand the treatment scope to a larger number of hematomas, so as to benefit more patients. To this end, we propose a new regimen of atorvastatin combined with low-dose dexamethasone and compare it in this study with the original regimen of atorvastatin alone. The two purposes of this study are: 1. To evaluate the efficacy of the new regimen combined with atorvastatin and dexamethasone in the treatment of CSDH, and whether it is superior to the original regimen; 2. To evaluate the safety of the combined regime.

The study is expected to enroll 240 patients with CSDH. The treatment period is 28 days, and the follow-up period is 180 days, with a total of 7 visits. You may need additional follow-up if your doctor deems it necessary.

## 3. What are the contents of this study?

The study is a multi-center, randomized, double-blind, placebo-controlled clinical study. If you agree to participate in the study and meet all the criteria for inclusion, you will be randomly assigned to one of the two treatment groups at a 1:1 ratio: treatment group (Atorvastatin combined with dexamethasone treatment group) 120 subjects and placebo group (Atorvastatin combined with dexamethasone placebo treatment group) 120 subjects.

| Group | Usage and Dosage |
|---|---|
| Atorvastatin combined with dexamethasone treatment group | Take a tablet containing 20 mg of atorvastatin calcium (Lipitor) QD for 4 weeks. Then dexamethasone acetate will be orally administered according to the following regimen: take dexamethasone acetate tablet 0.75mg, TID, 1 tablet at a time for the 1$^{st}$ and 2$^{nd}$ week; take dexamethasone acetate tablet 0.75mg, BID, 1 tablet at a time for the 3$^{rd}$ week; take dexamethasone acetate tablet 0.75mg, QD, 1 tablet at a time for the 4$^{th}$ week. |
| Atorvastatin combined with dexamethasone placebo treatment group | Take a tablet containing 20 mg of atorvastatin calcium (Lipitor) QD for 4 weeks. Then dexamethasone acetate placebo will be orally administered according to the following regimen: take dexamethasone acetate placebo tablet, TID, 1 tablet at a time for the 1$^{st}$ and 2$^{nd}$ week; take dexamethasone acetate placebo tablet, BID, 1 tablet at a time for the 3$^{rd}$ week; take dexamethasone acetate placebo tablet, QD, 1 tablet at a time for the 4$^{th}$ week. |

The study consisted of three phases: screening, treatment, and follow-up.

Screen period (Visit 0): First of all, you need to sign the informed consent. The study physician will then: record demographic data, medical history, and combined medication history; Measure your height and weight; Collect your vital signs (blood pressure, pulse rate and temperature, respiratory rate) and give you a physical examination. The investigator will evaluate your neurologic signs and symptoms, and perform a head CT scan to calculate the hematoma volume. You will also need to undergo laboratory tests, including: blood routine, biochemistry, coagulation function, and electrocardiogram. The above examination requires the collection of about 10ml blood (3 tubes in total).Among them, the blood routine, blood biochemistry, urine routine and blood coagulation function examination should not be conducted 7 days before your enrollment. In order to avoid unnecessary repeated blood sampling, this study accepts your blood sampling results before signing the informed consent. The inclusion/exclusion criteria will be reviewed by the study physician upon receipt of the results to verify that you meet the study criteria, and randomization will be completed.

Treatment period (The 1$^{st}$~4$^{th}$ visit): During the treatment period, eligible patients are required to receive the study drug according to the protocol and receive evaluation. Medication will start immediately after enrollment and continue for 28 days. The investigator will assess your status on the 1$^{st}$、7$^{th}$、14$^{th}$ and 28$^{th}$ day.

The 1st visit will record your vital signs; Distribution, use and recovery of experimental drugs; Record the combination of medication, surgical referral and all adverse events/adverse reactions/serious adverse events; Assess the patient's neurological symptoms and signs.

The 2nd visit will be completed as follows:Record your vital signs; Distribution, use and recovery of experimental drugs; Record the combination of medication, surgical referral and all adverse events/ adverse reactions/serious adverse events; Assess the patient's neurological symptoms and signs.

The 3rd visit will be completed as follows: Vital signs will be recorded; Head CT examination to calculate hematoma volume; Laboratory tests: blood routine, blood biochemistry, coagulation function, electrocardiogram; Distribution, use and recovery of experimental drugs; Record the combination of medication, surgical referral and all adverse events/adverse reactions/serious adverse events; Assess the patient's neurological symptoms and signs.

The 4th visit will be completed as follows: Vital signs will be recorded; Head CT examination to calculate hematoma volume; Laboratory tests: blood routine, blood biochemistry, coagulation function, electrocardiogram; Distribution, use and recovery of experimental drugs; Record the combination of medication, surgical referral and all adverse events/adverse reactions/serious adverse events; Assess the patient's neurological symptoms and signs; Evaluate neurological function scores.

Follow-up period (The 5th and 6th visit): During the follow-up period, the investigator will assess your status on 90th and 180th day without further use of the study drug.

The 5th visit will be completed as follows: Head CT examination to calculate hematoma volume; Record the surgical referral and all adverse events/adverse reactions/serious adverse events; Assess the patient's neurological symptoms and signs; Evaluate neurological function scores.

The 6th visit will be completed as follows: record the surgical referral and all adverse events/adverse reactions/serious adverse events; Assess the patient's neurological symptoms and signs; Evaluate neurological function scores.

All tests you perform at each visit are designed to help your physician determine if you are still eligible to participate in the study and to ensure your safety.

If the study is completed or terminated early, you will not receive any study drug treatment. At the same time, for your safety, you will be scheduled for physical examination, weight and

vital signs measurement, electrocardiogram examination and laboratory examination after finishing the study early.

### 4. What do I need to do during the study?

If you choose to participate in this study, please cooperate well with the investigator during the study and abide by the following rules:

①Timely completion of follow-up; Measure fasting blood in the morning of $0^{th}$ , $3^{rd}$ , and $4^{th}$ visit.

②Please inform your physician of any discomfort during the trial so that he can deal with it accordingly.

③You will need to provide your physician or investigator with information about your health during the study, especially any changes that may be beneficial or detrimental. If you and your spouse discover pregnancy during the course of the study, inform your physician immediately.

④ If you seek medical advice from other physicians, please inform them that you are participating in this clinical trial.

⑤In accordance with the study protocol, you are not allowed to use glucocorticoids, growth hormones, sex hormones and other drugs that may potentially affect angiogenesis during the study medication.

⑥It is important for you to follow your physician's instructions regarding the medications and treatment for this study. During the trial, regardless of which treatment you receive and whether you think it is related to the study drug, you are expected to inform us of all the symptoms that occur. Please inform us of any increase or change in the use of conventional drugs during the study period.

### 5. Are there any alternative treatment options?

If you do not want to participate in this study, you may have surgery or other conservative treatment. If you decide not to participate in this study, your physician will continue to advise you on the treatment options that are appropriate for you.

If you do not meet all study inclusion criteria, your study physician will decide how to proceed with treatment.

### 6. Study-related damage

Side effects of atorvastatin depend on dose, duration, and frequency of use. The most common adverse events are constipation, flatulence, indigestion and abdominal pain. The more serious complications are elevated transaminase and rhabdomyolysis. Transaminase elevation, which is not severe, can be found in liver function tests and it usually returns to normal 1 to 2 weeks after drug withdrawal. Statins have been used to treat thousands of cases of CSDH, but there

has been no confirmed case of rhabdomyolysis. The incidence of these two side effects is very low and both are reported to be completely alleviated after drug withdrawal.

Previous literature demonstrate a lot of side effects of dexamethasone, mainly including edema, obesity, high blood, digestive tract ulcer and the increased risk of pneumonia, but the side effect refers to the large doses and long-term use of dexamethasone. Low-dose dexamethasone is used in this study and the course of treatment is only 4 weeks. Previous trials have shown that the associated side effects after treatment are rare in patients. The main dexamethasone-related side effects observed include increased appetite, slightly increased weight, and slightly increased blood sugar. There is no serious complication.

These side effects will be carefully detected and treated by your physician.

During the study, you need to take 3 blood tests with total volume of appropriate 30ml. Although blood drawing is routine, it may cause discomfort and petechiae at the site of acupuncture. The injection will cause slight pain, bruising and swelling

There may be other side effects or risks that are not yet clear. If you experience any discomfort during the study, please contact your research physician immediately. Your research physician will provide you with appropriate medical treatment and necessary treatment.

**7. Study-related compensation**

If you have adverse events from the beginning to the end of the study, it shall be determined by the investigator whether the adverse event is related to the study drug or the diagnostic examination required by the study protocol. If you are injured caused by the effects of this treatment in the study, your treating physician and hospital will provide you with all necessary treatment. Treatment will be funded in accordance with the requirements of Chinese clinical trials.

If you have any questions at any time, please feel free to contact your physician or study coordi nator/nurse as soon as possible, at the contact information is：________________________.

If you have any questions or complaints about the implementation of this study, you may conta ct the Ethics Committee at the by telephone ,the phone number is：________________________

**8.Possbile benefits？**

Since both methods in this study have been preliminarily proven to be effective, and atorvastatin alone has been demonstrated to be effective in a randomized controlled clinical

trial, it is possible to achieve a “cure” without surgical treatment via participating in this clinical observation.

Once participating in this study, you will be closely contacted by a professional medical team who will closely observe the changes in your condition. You can get more medical information and helpful medical help and advice, so as to bring additional medical convenience and improve medical efficiency.

Once your hematoma grows and requires surgery, we ensure that you will be operated on by a senior doctor to ensure the quality and safety of the operation.

**9. If there is any new progress/information?**

We will keep you informed of any new information regarding the study drug, that may influence your decision to continue participating in the study.

Please note that the results of this study may be published in medical journals, but you will not be identified.

**10. Will all my personal information remain confidential?**

By signing this informed consent form, you have consented to the collection and use of your personal data (" Study Data ") by the study physician and the staff of the Research Center for the study. This includes your date of birth, gender, race, and medical condition.

Your informed consent for the use of the study data does not set a specific deadline, but you may revoke your informed consent by notifying your study physician at any time.

Your information will be kept confidential at all times. Only the research physician will retain your basic information, and your initials and code will be used in other documents of the study. In addition to your research physician, representatives of the sponsor of this study, supervisors, inspectors, representatives of the Ethics Committee and the China Food and Drug Administration may consult your original medical data related to the study to ensure that the study is standardized and the data are authentic and reliable. All information will be kept confidential and the results of this study may be published in medical journals, but your identity will not be disclosed.

By signing this document, you agree and allow the research team to share your health data with government agencies, Ethics Committee (which oversee the study), the sponsors, and those working for the sponsors.

However, the sponsor and those who work for the sponsor will not be able to identify you by name. The health data they receive will contain only your initials, date of birth, and date of study visit. Your name will not appear in any published report on the study, or in any other scientific publication or newsletter.

You may revoke your permission to use and share your health data with the research physician at any time. If this happens, you will not be able to continue participating in the study. From the date you revoke your license, we will no longer collect new identifiable health data. But we will use your health data that has been collected previously.

**11. Principle of trial**

Your participation in this study is entirely voluntary. You have the right to withdraw from the study at any time without discrimination or retaliation, and your medical treatment and rights will not be affected. You have the right to know about the possible adverse effects of the study drug. If your continued participation in the study causes harm to your health, if your health is no longer fit to continue the study, or if you are unable to comply with the requirements of the study protocol, your physician may terminate your study without your consent. If you decide to withdraw from the study, please be sure to contact the physician in charge of the study, who will arrange for a discontinuation of the visit for evaluation of the various indicators.
The sponsor of this study may also suspend or restrict the entire study early for safety or other reasons.

**12. Where should I resort to if I get hurt?**

If you have any questions regarding this study, or if any research-related injury occurs, please contact the study physician (investigator) by:
Investigator Name：________________ Contact Number：____________________

If you have any questions about your rights as a study subject, please contact the ETHICS Committee by:
Ethics Committee contact person Name：________________ Contact Number：_________
__________

## Informed consent signing page

**I have fully understood all the contents contained in this consent form and the risks and benefits that may arise from this study. My questions have been satisfactorily answered and I am volunteering to participate in this study.** I agree to follow up on time in the study, and to receive relevant examinations related to this study. I will comply with the subject's instructions and fully cooperate with the investigator to truthfully and objectively provide the health status and relevant information before, during and during the follow-up period of the study. By informing the study physician, I can choose not to participate in the study or withdraw from the study at any time without penalty or loss of any of the benefits I would otherwise have enjoyed. I understand that the investigator has the right to terminate the study at any time according to my condition. I understand that I will receive a signed and dated copy of this consent form. I agree to collect, use and share my health data in this study. After careful consideration, I decide to volunteer for the "Efficacy and safety of atorvastatin combined with dexamethasone in a multicenter, randomized, double-blind, placebo-controlled clinical study of chronic subdural hematoma."

| | | | |
|---|---|---|---|
| **Subject's Signature regular script** | ___________________ | **Subject's Signature** | ___________________ |
| | | **Date (year/month/day)** | ___________________ |

**Legal representative**（If applicable）：

| | | | |
|---|---|---|---|
| **Legal representative's signature regular script** | ________________ | **Legal representative's signature** | ___________________ |
| **Relationship with subjects** | ________________ | **Date (year/month/day)** | ___________________ |
| **Reasons for signature of legal representative：** ______________________________________________ | | | |

**Observer**（If applicable）

| | | | |
|---|---|---|---|
| **Observer 's signature regular script** | ________________ | **Observer's signature** | ___________________ |
| | | **Date (year/month/day)** | ___________________ |
| **Reasons for signature of the observer：** ______________________________________________ | | | |

I have explained the study in detail to the above subject and provided him/her with a signed and dated copy of this consent form.

| | | | |
|---|---|---|---|
| **Investigator's Signature** | ___________________ | **Investigator's Signature** | ___________________ |

**regular script**

**Date(year/month/day)** ____________________
